# The Effectiveness of Digital Interventions to Increase Preventive Care Uptake in Older Adults: Systematic Review

**DOI:** 10.2196/83446

**Published:** 2026-04-29

**Authors:** Lindsay Burton, Kathy L Rush, Mindy A Smith, Robert Janke

**Affiliations:** 1 School of Nursing, University of British Columbia, 1147 Research Road, Kelowna, BC, V1V 1V7, Canada, 1 250 807 9561; 2 Department of Family Medicine, Michigan State University, East Lansing, MI, United States; 3 Library, University of British Columbia, Kelowna, BC, Canada

**Keywords:** older adults, preventive care, digital health, systematic review, screening, vaccination

## Abstract

**Background:**

Older adults face increasing health risks associated with aging and chronic disease; yet, uptake of recommended clinical preventive services remains low. Digital health interventions have the potential to enhance access and engagement, but their effectiveness in older adult populations remains unclear.

**Objective:**

This systematic review aimed to examine the range and types of digital clinical preventive service interventions and assess their impact on preventive care uptake among older adults.

**Methods:**

We conducted a systematic review of peer-reviewed research literature published since 2014. Eligible studies included experimental and quasi-experimental designs evaluating digital interventions targeting community-dwelling adults aged 60 years and older. Interventions focused on high-priority preventive services, including cancer screening and adult immunizations. Data were extracted using a standardized form and synthesized narratively due to heterogeneity in study designs and outcomes.

**Results:**

In total, 24 studies involving over 1.3 million participants from 11 countries were included. Interventions used a range of digital tools, including telephone calls, SMS text messages, patient portals, and video-based education. While some digital and automated interventions demonstrated modest improvements in preventive services uptake, results were mixed. Interventions incorporating personalized elements (eg, tailored telephone counseling or in-person education) were generally more effective than generic, automated communications. Few studies reported on digital literacy support or intervention reach, and engagement with digital platforms was often low.

**Conclusions:**

Digital interventions can support modest improvements in preventive services uptake among older adults, particularly when personalized or combined with human interaction. However, assumptions of digital fluency and limited reporting on engagement constrain generalizability. Future research should prioritize inclusive design, detailed reporting, and strategies that address digital equity to better support older adult populations.

## Introduction

Over the next decade, the percentage of Canadian people aged 65 years and older is projected to increase from 18.5% in 2021 to 22.9% by 2030. Similarly, the proportion of individuals aged 85 years and older is projected to increase from 2.3% in 2021 to 4.6% by 2050 [1]. Accelerated population aging, coupled with increasing chronic disease prevalence, has driven rising health care use and costs [2-5]. For instance, the average per-person health care spending for Canadian people aged 65+ years (US $9260) is 4 times higher than those <65 years (US $2083) [6]. Similarly, older adults in the United States represent 13.5% of the population but account for 45.2% of the top 10% of health care spenders [5]. The trend of population aging and estimates of increased costs highlight the urgent need for approaches that promote healthy aging, reduce chronic disease, and optimize quality of life for older adults.

Engaging older adults to stay current on recommended clinical preventive services is a proactive approach to promote healthy aging. Preventive services comprise activities aimed at disease prevention (eg, immunizations and alcohol or tobacco cessation) and early disease detection (eg, cancer screening, blood glucose, and blood pressure monitoring) [7]. Guidelines for recommended preventive services based on age, gender, and other risk factors have been developed using best evidence for cost-effectiveness and reduced morbidity and mortality [8,9]. However, despite national and provincial preventive services guidelines and recommendations, few older adults are up to date on all recommended preventive activities. The number of recommended preventive services and their importance increases with age; yet, completion rates are alarmingly low for older adults. According to the British Columbia Cancer Agency, only 52% of women aged 50‐74 years are screened for breast cancer compared to a target of 70% [10]. A US study estimated a completion rate for all relevant clinical preventive services of only 7.9% for older adults [11].

The rapid development and implementation of digital health services, including preventive services, during and after the COVID-19 pandemic have increased access and convenience [12] but have also had the untoward impact of excluding individuals with limited digital literacy. Although older adults are less frequent users of technology compared to younger age groups [13], evidence indicates that older adults’ willingness to engage with digital technologies is growing steadily [14]. However, there remain challenges and barriers for older adults when accessing digital resources and health services (eg, lack of support and poor functionality) [15,16], which contribute to digital inequity and hinder older adult engagement in digital preventive services. Understanding the current state of the science regarding the effectiveness of digital interventions for preventive service uptake in older adults would provide valuable, evidence-based insights to better leverage technology in meeting their needs.

Although systematic reviews have synthesized evidence focused on individual preventive services, such as breast [17] and colon cancer [18] screening, no comprehensive review has been conducted looking specifically at older adults and digital aspects of preventive care services. As such, this review aims to answer this question: What are the range and types of digital clinical preventive services interventions (intervention), and what is their impact on older adults’ (population) uptake of clinical preventive services (outcome)?

## Methods

### Design

This systematic review was designed following the JBI Manual for Evidence Synthesis [19,20] and the Cochrane Handbook for Systematic Reviews of Interventions [21].

### Search Strategy

This systematic review captures peer-reviewed research papers addressing digital preventive services uptake targeting older adults. The digital tools considered included telephone (automated or synchronous), text messaging, web-based educational materials or materials delivered via video, or patient portal. The search strategy was developed in consultation with a health sciences librarian (RJ; see Multimedia Appendix 1 for the search strategy from Embase). Search terms were selected based on the review question’s main content areas of older adults and clinical preventive services and included a combination of keywords and subject headings. Databases used in the search included MEDLINE, CINAHL, Embase, and PsycINFO. The search strategy was modified only slightly between the databases to incorporate their specific subject headings. Database searches were conducted on December 11, 2023, and updated on July 13, 2025.

### Inclusion and Exclusion Criteria

Eligible literature included experimental (eg, randomized controlled trials) and quasi-experimental study designs. Qualitative and observational studies that did not test an intervention were excluded. Studies were included if they addressed preventive services uptake among older adults in response to single (eg, breast cancer screening, colorectal cancer, and blood pressure) or multiple services (eg, blood pressure, cancer screening, and seasonal vaccination). Interventions were limited to high-priority preventive care services based on high clinically preventable burden and established immunization schedules for older adults [8,22]. Preventive care services included 5 related to screening (cardiovascular disease, type 2 diabetes mellitus, hypertension, colorectal cancer, and breast cancer) and 6 related to vaccines (seasonal influenza, pneumococcal polysaccharide, shingles, COVID-19, and tetanus and or diphtheria).

Studies were eligible if they included samples that were primarily community-dwelling older adults. Because some studies did not report disaggregated age and used broad age categories (eg, “older adults” defined as 55+ years), we applied the following rules: studies in which all participants were aged 60 years or older were considered eligible; and if participants younger than 60 years were included, at least 50% of the sample had to be aged 65 years or older. This approach ensured consistency with our focus on older adults while allowing inclusion of studies with varied reporting practices. Studies were excluded if they focused on subpopulations (eg, familial risk and rheumatoid arthritis) or noncommunity-dwelling older adults (eg, long-term care residents) because they do not reflect the asymptomatic population for which preventive services guidelines are designed. Publications were limited to the last 10 years from when the review began to capture current strategies [9,22]. Studies were excluded if they did not report the results of primary research (ie, editorials, letters, search and research protocols, or systematic reviews).

### Paper Selection

Two reviewers (LB and MAS) completed abstract screening and full-text screening, blinded to each other’s decisions. Search results were added to the abstract management software Covidence for deduplication. A small sample of 10‐15 records was tested against the stated inclusion and exclusion criteria [23]. The initial test of the screening process involved calibrating agreement, clarifying criteria, and making any necessary modifications before full screening. Following screening calibration, the remaining screening was conducted independently by the 2 reviewers (LB and MAS) [23]. Conflicts were assessed and resolved using consensus by 2 reviewers (LB and MAS). Interrater reliability was calculated using Cohen κ within Covidence software. The Cohen κ for abstract screening was 0.68, indicating substantial agreement [24].

### Quality of Evidence

Quality of evidence was assessed using the Cochrane revised tool to assess risk of bias in randomized trials (Risk of Bias 2 [RoB 2]) [25] and the Risk of Bias in Non-randomized Studies—of Interventions (ROBINS-I) tool [26]. Both tools concentrate on the study’s internal validity [25,26]. In both tools, bias refers to a tendency for the results to deviate systematically from what would be expected from a randomized trial, conducted on the same participant group with no flaws in its execution [25,26]. RoB 2 assesses 5 domains of bias: (1) randomization process, (2) deviations from intended interventions, (3) missing data, (4) measurement of outcomes, and (5) selection of reported results [25]. For the RoB 2 assessment, each domain is assigned a risk of bias assessment of low, some concerns, or high risk of bias [25]. ROBINS-I assesses six domains of bias: (1) confounding, (2) selection of participants, (3) classification of interventions, (4) missing data, (5) measurement of outcomes, and (6) selection of reported results [26]. For the ROBINS-I assessment, each domain is assigned a risk of bias assessment of low, moderate, serious, or critical [26]. An overall risk of bias assessment for each paper is then conducted for both RoB 2 and ROBINS-I tools based on the assessment of the domains [25,26]. Risk of bias assessments were conducted by 1 team member and verified by a second reviewer.

### Data Extraction and Analysis

Data extraction was conducted using a standardized form. Elements for extraction included study characteristics, study population, intervention characteristics, type of preventive service targeted, single or multiple services, and change in preventive care uptake. Preventive services uptake was defined as the completion of a screening test or immunization, either by self-report, review of medical chart, or insurance claims. Data extraction was conducted by 1 research assistant and confirmed by 2 additional reviewers (MAS and LB).

Data were synthesized using a narrative approach to explore the effectiveness of digital preventive services interventions for older adults. This method was suitable, given the variability in designs and interventions among the studies analyzed [27]. Technology complexity was determined using a researcher-developed scale of low to high based on the degree of interactivity required of older adults to receive the preventive-services intervention [28]. Scale and rating criteria are detailed in Multimedia Appendix 2. The uptake of clinical preventive services was compared across different intervention strategies to identify potential patterns and insights. Data heterogeneity prevented meta-analysis.

## Results

### Overview

**Figure 1.**
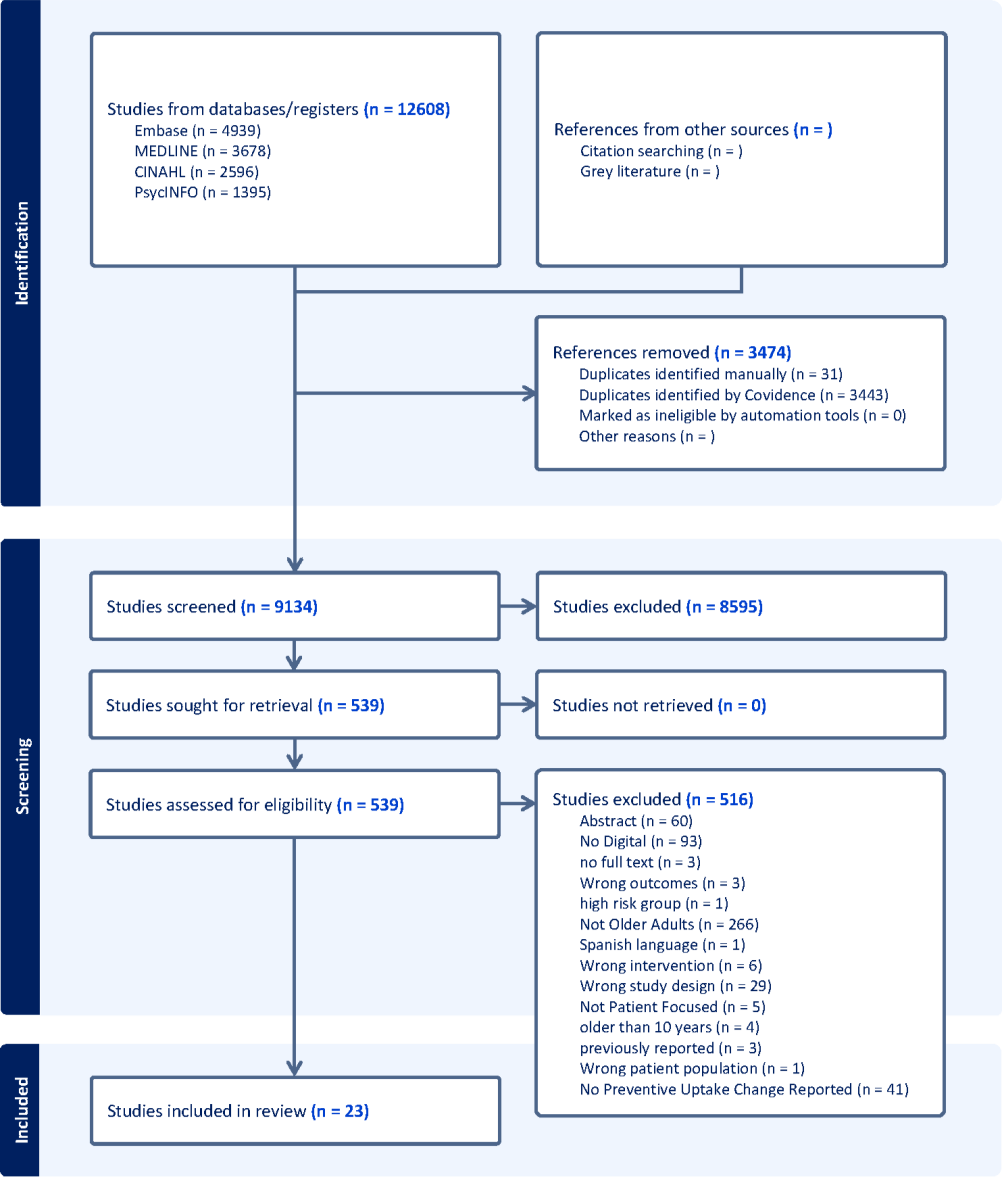
Summary of literature search and study selection process.

The studies included in this review (n=23) represented 1,205,652 participants from 11 countries: the United States (n=11), Spain (n=3), Hong Kong (n=2), and 1 each from Australia, China, Denmark, France, Israel, Lebanon, and the United Kingdom. Studies were published between 2015 and 2025. A flowchart summarizing the search, screening, and study selection process is included in Figure 1 [29]. Designs included randomized controlled trials (n=20) and quasi-experimental studies (n=3). The majority (n=14) of included studies had a low risk of bias; the remaining had some or unknown risk of bias, but this was not a concern for the conclusions drawn by each study. The preventive services targeted in the interventions included colorectal cancer screening (n=10), influenza vaccination (n=6), breast cancer screening (n=3), pneumococcal vaccination (n=2), combined vaccinations (n=1), and COVID-19 vaccination (n=1; Table 1).

**Table 1.** Summary of included papers.

Study	Country	Design	Risk of bias	Sample	Targeted preventive care service	Simple intervention description	Change in preventive service uptake
Basch et al (2015) [30]	United States	RCT^a^	Low	564 participants, average age 57.6 (SD 4) years, 69.3% women, 17.6% White.	CRC^b^ screening	Comparison: CRC print education material Intervention AD^c^: Comparison+AD Intervention TTE^d^: Comparison, AD+targeted telephone education	Among those 60 years of age or older, AD plus telephone education group had a higher screening rate than the print group (27.3 vs 7.7%; *P*=.02).
Chan et al (2015) [31]	Hong Kong	RCT	Some concern—lack of detail of concealment	2517 participants aged >65 years, 57.3% women; ethnicity not reported.	Pneumococcal vaccination	Comparison: Standard of care Intervention: Education telephone intervention	1325 (53%) participants received the polysaccharide vaccine injection as recorded in the clinical management system—716 (57%) in the intervention group and 609 (48%) in the control group.
Denis et al (2017) [32]	France	RCT (3 arm)	Low	5691 participants, average age 61.2 (SD 6.8) years, 49.4% women; ethnicity not reported.	CRC screening	Comparison: FOBT^e^ kit Intervention CATI^f^: CATI and FOBT kit Intervention MI^g^: Motivational telephone interview and FOBT kit	Only 5691 (19.9%) people were actually counseled. There was no difference in completion between the intervention groups taken together and the control group (13.9% vs 13.9%) in intention-to-treat analysis.
Fernández et al (2015) [33]	United States	RCT (3 arm)	Some concern—outcome measure self-reported	656 participants, age 60 to 69 years: 31.8%, age 70+ years: 20.3%, 69.3% women, 100% Hispanic or Latino or Mexican.	CRC screening	Comparison: No intervention Intervention SMPI^h^: SMPI with DVD and flipchart Intervention TIMI^i^: TIMI delivered on tablet computers	In the intention-to-treat analysis, 13.6% in the SMPI group, 10.2% in the TIMI group, and 10.8% in the comparison group completed CRC screening (not statistically significant).
Fleming et al (2018) [34]	United States	Quasi-experimental design	Moderate concern	3415 participants, average age 60.0 (SD 6.3) years, 50.5% women, 32.8% White.	CRC screening	Comparison: Mass-mailing FIT^j^ kit Intervention phone: Comparison+telephone or recorded message Intervention in-person: Comparison+one-on-one conversations in clinic	One-on-one conversations either in person (OR^k^ 24.63, 95% CI 19.28‐31.46) or via telephone or recorded message (aOR 14.74, 95% CI 10.96‐19.82) were more effective for completing the at-home CRC screening than mass mailing before receiving the FIT.
Ghadieh et al (2015) [35]	Lebanon	RCT (6 arm)	Low	350 participants aged 60+ years not receiving the vaccine, 54.9% women; ethnicity 100% Chinese.	Pneumococcal vaccination	Intervention: Telephone reminder±education Intervention: Email reminder±education Intervention: SMS text message reminder±education	Vaccination rates increased to 16.5% in short phone call group, 7.2% in SMS text group, and 5.7% in the email group; from 17.2% to 20.4% in patients older than 65 years.
Hagoel et al (2016) [36]	Israel	RCT	Low	48,091 participants, average age 60.44 (SD=6.04) years, 51.1% women; ethnicity not reported.	CRC screening	Comparison: No intervention Intervention interrogative: Interrogative SMS text message reminders±social context Intervention noninterrogative: Noninterrogative SMS text message reminders±social context	At 6 months, interrogative (9.8%; *P*=.002), interrogative-with-social-context (10.3%; *P*<.001), and noninterrogative-with-social-context (9.6%; *P*<.009) messages had greater uptake compared to no messages in control (8.5%).
Hirst et al (2017) [37]	United Kingdom	RCT	Low	176,231 participants, 43.5% aged 60‐70+ years, 53% women, 51% White.	CRC screening	Comparison: Usual care Intervention: SMS text message reminders	Uptake was 40.5% in the intervention group and 39.9% among controls. Uptake did not differ significantly between groups of older adults (aOR 1.03, 95% CI 0.94‐1.12; *P*=.56) but did vary for first-time invitees (uptake 40.5% in intervention vs 34.9% in the control group; aOR 1.29, 95% CI 1.04‐1.58; *P*=.02).
Hurley et al (2018) [38]	United States	Pragmatic RCT	Low	8269 participants, median age 66 years, 52% women; ethnicity not reported.	Influenza or tetanus, diphtheria, acellular pertussis vaccine	Comparison: Usual care Intervention: Up to 3 reminders or recalls	The intervention was associated with receipt of any needed vaccine in the adults aged ≥65 years (aOR 1.15, 95% CI 1.02‐1.30).
Jiang et al (2022) [39]	China	RCT	Low	15,769 total participants, 616 aged 65+ years, 63.3% women; ethnicity not reported.	Influenza vaccination	Comparison: No intervention Intervention: Video-led educational intervention	Intervention group more willing to receive influenza vaccination than control group (64.6% vs 51.4%; *P*<.05). Vaccination uptake rate was higher in the intervention group (10.3% vs 3.4% among controls; OR 3.23, 95% CI 1.25‐8.32; *P*<.001).
Lieu et al (2022) [40]	United States	RCT (3 arm)	Low	8287 participants not receiving the vaccine after prior outreach, average age 72.6 (SD 7) years, 56% women 100% Black or Latino.	COVID-19 vaccinations	Comparison: Usual care Intervention: Electronic and/or mail outreach from the provider with or without culturally tailored content	Higher COVID-19 vaccination rates for intervention with cultural content versus usual care (24% vs 21.7%; aHR^m^ 1.22, 95% CI 1.09‐1.37), and for primary care provider outreach without cultural content (23.1% vs 21.7%; aHR 1.17, 95% CI 1.04‐1.31).
López-Torres Hidalgo et al (2016) [41]	Spain	RCT (4 arm)	Some concern—self-reported outcome	1129 participants, average age 61.4 (SD 6.6) years, 60.1% women; ethnicity not reported.	CRC screening	Comparison: No intervention Intervention: Written, telephone, or face-to-face recommendation of CRC screening	Uptake was 15.4% (OR 2.32, 95% CI 1.23‐4.37) in the written information group, 28.8% (OR 4.38, 95% CI 2.38‐8.07) in the telephone information group, and 8.1% (OR 1.26, 95% CI 0.59‐2.68) in the face-to-face group versus 5.9% in the control group.
Luckmann et al (2019) [42]	United States	RCT (3 arm)	Some concern—unclear description of randomization	30,160 participants, 52.3% aged 60‐84 years, 100% women, 73% White.	Breast cancer screening	Intervention letter: Reminder letter only Intervention reminder: Letter plus reminder call Intervention counseling: 2 letters and tailored education and MI	Over 4 years, mammography adherence was highest with a reminder call (83%) versus counseling (80.8%) or letter only (80.8%), *P*=.03.
Luque Mellado et al (2019) [43]	Spain	Quasi-experimental study	Moderate concern	2343 participants, 53.4% women; age and ethnicity not fully reported.	CRC screening	Telephone interview	Participation increased with age for both genders (60‐ to 64-year group: OR 1.55, 95% CI 1.35‐1.78 and 64‐ to 69-year group: OR 2.07, 95% CI 1.80‐2.38).
Modin et al (2023) [44]	Denmark	RCT (9 arm; usual care or 9 electronic letters with designs based on behavioral concepts)	Low	964,870 participants, average age 73.1 (SD 6.2) years, 27.4% had CVD^n^, 52% women; ethnicity not reported.	Influenza vaccination	Comparison: Usual care Intervention arms: 9 different electronic nudges emphasizing the potential cardiovascular benefits of influenza vaccination	Influenza vaccinations were received by 83.1% of participants with CVD versus 79.2% of participants without CVD (*P*<.001). Compared with usual care, a letter intervention increased vaccination rates for those with (absolute difference,+0.60 percentage points; 99.55% CI –0.48 to 1.68) and without CVD (+0.98 percentage points; 99.55% CI 0.27‐1.70; *P* for interaction .41). Only the repeated letter strategy with a reminder follow-up letter 14 days later was effective in increasing influenza vaccination, irrespective of CVD.
Regan et al (2017) [45]	Australia	RCT	Low	12,354 total participants, 3613 aged 65+ years, 52.6% women, 48.6% non-Indigenous.	Influenza vaccination	Comparison: No reminder Intervention: SMS text message reminders	For those ages 65+ years, 20.5% in the intervention group versus 15.8% in the control group were vaccinated (absolute difference +4.7; RR^o^ 1.26, 95% CI 1.10‐1.45; NNT^p^ 21).
Sepucha et al (2023) [46]	United States	RCT	Some concern—not all intervention participants received decision coaching	800 participants, average age 60 (SD=8) years, 53% women, 73% White.	CRC screening	Comparison: Usual care Intervention: Decision aids for shared decision-making (SDM) and decision coaching	Intervention respondents reported higher SDM scores (mean difference 0.7; 95% CI 0.4‐0.9; *P*<.001) and less decisional conflict than controls (−21%; 95% CI −35% to −7%; *P*=.003).
Szilagyi et al (2020) [47]	United States	RCT (4 arm)	Low	164,205 participants, 18.7% aged 65+ years, 58% women, 57% White.	Influenza vaccination	Comparison: No portal reminder Intervention arms: Patient portal reminders provided one, two, or three times	The adjusted risk ratio for influenza vaccination among individuals aged 65 years and older was 0.97 (95% CI 0.92‐1.02), with no dose response by number of reminders sent for all age groups.
Szilagyi et al (2022) [48]	United States	RCT (6 arm)	Low	196,486 total participants, 29,795 aged >65 years without diabetes, 58.5% women, 56.9% White.	Influenza vaccination	Comparison: No messages Intervention arms: Personalized messages (5 types around loss or gain frame) sent by a health system’s patient portal or reminder alone	Influenza vaccination rates for older adults were 55.6% with no statistically different vaccination rates for any study group versus control.
Venishetty et al (2023) [49]	United States	Prospective, pragmatic, two-arm intervention study	Low	344 participants, average age 60.1 (SD 5.5) years, 68% women, 89% White.	CRC screening	Comparison: Mailed FIT kit and follow-up call Intervention: Mailed FIT kits with introductory phone calls by a patient navigator or community health worker; there was variability in FIT kits distributed by health clinics	Overall, 25.3% of participants returned the FIT kit. The return rate was higher for the intervention group versus the usual care group (27.8% vs 22.8%; *P*=.003). Participants who received a 1-day sampling kit had a higher return rate (31.3% vs 23.3%).
Vives et al (2024) [50]	Spain	RCT	Low	10,433 participants, average age 60.3 (SD 5.3) years.	Breast cancer screening	Comparison: Invitation letter+SMS text message reminder Intervention: SMS text message invitation to mammography with a timed appointment+SMS text message reminder	No difference in the screening participation rate within 12 weeks of the invitation in the SMS text message group (86.6%) and in the letter group (87.3%).
Wang et al (2023) [51]	Hong Kong	RCT	Some concern—self-reported outcome	396 participants, average age 70.2 (SD=4.3) years, 63% women; ethnicity not reported.	Influenza vaccination	Comparison: Standard online video Intervention: Chatbot-delivered online intervention tailored to the stage of change once every 2 weeks for 4 sessions	Influenza vaccination rate was higher in the intervention group than the control group at month 6 (50.5% vs 35.3%; *P*=.002).
Wu and Lin (2015) [52]	United States	RCT	Some concern—self-reported outcome	193 participants, 100% women, 100% Chinese American.	Breast cancer screening	Comparison: Mammography brochure Intervention: Tailored telephone counseling to personal barriers	For women aged ≥65 years, there was a significant difference in uptake between intervention and control groups (51% vs 25%).

a RCT: randomized controlled trial.

b CRC: colorectal cancer.

c AD: academic detailing.

d TTE: tailored telephone education.

e FOBT: fecal occult blood test.

f CATI: computer-assisted tailored telephone interview.

g MI: motivational interview.

h SMPI: small media print intervention.

i TIMI: tailored interactive multimedia intervention.

j FIT: Fecal Immunochemical Test.

k OR: odds ratio.

l aOR: adjusted odds ratio.

m aHR: adjusted hazard ratio.

n CVD: cardiovascular disease.

o RR: relative risk.

p NNT: number needed to treat.

Digital tools used included the single or combined use of telephone calls, text messages, patient portal messages, email, and videoconferencing. No studies included a description of digital tool support or training for participants. Under half of the studies (n=9) included a description of the digital intervention’s reach (ie, messages opened and calls answered; Table 1).

### Range of Interventions

The analysis revealed a wide range of approaches aimed at improving patient engagement and adherence to preventive health screenings and vaccinations. Three main categories of interventions were classified: digital and automated communication, personalized and tailored communication, and in-person interventions supplemented with digital supports.

#### Digital and Automated Communication Interventions

To increase vaccination and screening rates, 12 studies tested interventions that used automated digital communication tools including electronic health record portal messages, interactive automated calls, automated text messaging, and 1 chatbot-integrated messaging intervention [32,35-38,40,44,45,47,48,50,51]. Across the 12 included trials, most interventions used low-complexity, system-integrated technologies, with a smaller subset relying on simple stand-alone channels (Table 2). Results were mixed, with only 4 interventions demonstrating significant improvements in vaccination or screening rates compared to usual care, as detailed below.

**Table 2.** Complexity of technology for digital and automated communication interventions.

Study	Targeted preventive care service	Simple intervention description	Technology	Digital intervention reach	Patient facing complexity
Denis et al (2017) [32]	Colorectal cancer screening	Tailored telephone—computer-assisted counseling or motivational interview	Telephone	33.6% of telephone counseling attempts were technically successful	Low
Ghadieh et al (2015) [35]	Pneumococcal vaccination	Patient reminder system	Telephone, email, or SMS text messages with or without education	2% (121/6,177) SMS transmission failures	Low
Hagoel et al (2016) [36]	Colorectal cancer screening	SMS text message reminders	SMS text messages	Not specified	Low
Hirst et al (2017) [37]	Colorectal cancer screening	Text message reminders	Text message	Mobile phone coverage varied by demographics: 42.7%‐53.3% had registered numbers; 73.4% (1,023/1,393) successfully received text reminders	Low
Hurley et al (2018) [38]	Influenza or tetanus, diphtheria, acellular pertussis vaccine	Centralized reminder or recall	Telephone	Not specified	Low
Lieu et al (2022) [40]	COVID-19 vaccinations	Electronic and/or mail outreach from provider with or without culturally tailored content or usual care	Electronic secure messages	Not specified	Moderate
Modin et al (2023) [44]	Influenza vaccination	Electronic nudges emphasizing potential cardiovascular benefits of influenza vaccination	Electronic letters	Not specified	Low
Regan et al (2017)[45]	Influenza vaccination	SMS text message reminders	SMS text messages	75.5% of eligible patients had mobile phone numbers in the EMR^a^	Low
Szilagyi et al (2020) [47]	Influenza vaccination	Patient portal reminders provided 1, 2, or 3 times or none (control)	Online patient portal messages	52.9% to 58.8% of intervention participants opened at least 1 portal reminder	Moderate
Szilagyi et al (2022) [48]	Influenza vaccination	Personalized messages (5 types around loss or gain frame) sent by a health system’s patient portal or reminder alone	Online patient portal messages	Not specified	Moderate
Vives et al (2024) [50]	Breast cancer screening	SMS text message invitation to mammography with a timed appointment	SMS text message	Not specified	Low
Wang et al (2023) [51]	Influenza vaccination	Chatbot-delivered online intervention tailored to the stage of change once every 2 weeks for 4 sessions or standard online video	Chatbot-delivered online intervention	Text messages failed to be delivered to 92 (1.7%) women in intervention	Low

a EMR: electronic medical record.

Of the 4 studies aimed at cancer screening, only 1 significantly improved uptake compared to usual care. Hagoel et al [36] conducted a randomized experiment comparing 4 text messages with different theory-based content to no messages (control) and found that 3 of the 4 messages significantly increased colorectal cancer screening rates compared to the control. Conversely, 2 interventions that used reminders, text messages [37], or phone calls [32], along with mailed Fecal Immunochemical Test (FIT) kits, found no difference in intervention groups compared to usual care (FIT kit plus 1 reminder letter). A breast cancer screening intervention found no difference between text message (intervention) and letter (control) invitations [50].

In 5 studies aimed at increasing seasonal influenza vaccination uptake rates, only marginal increases were observed. Even when increases were observed, absolute increases from baseline for different automated letters were small (0.73 and 0.89 percentage points, respectively) [44]. In the 2 studies examining generic reminders through a patient portal or via text messages, only marginal, nonsignificant increases in vaccination rates were found from baseline [45,47]. Another large-scale study used reminder messages sent through the health system patient portal, tailored to the health belief model loss or gain framework, to promote influenza vaccination uptake [48]. They similarly reported no significant increase in vaccination rates among older adults compared to generic digital reminders [48]. However, in 1 theory-based intervention that used a chatbot to deliver promotional videos corresponding to stages of change theory compared to generic messages, investigators found a significant between-group difference in vaccination rates at 6 months (50% of the intervention group vs 35% of the control group; *P*=.002) [51].

In total, 3 studies that examined interventions designed to increase the uptake of vaccinations showed small but statistically significant increases. One study compared 3 different reminder modalities to encourage pneumococcal vaccination: a standardized phone call reminder from a nurse, text messaging, or email [35]. Vaccination rates increased from 17.2% to 20.4%, with the largest uptake observed in the phone call group (16.5%), compared to text messaging (7.2%) and email (5.7%) [35]. Hurley et al [38] used a centralized vaccine reminder system for pneumococcal, tetanus, and influenza vaccinations, with the intervention group receiving up to 2 auto-dial phone calls followed by a postcard. Findings showed a significant increase in receiving any of the 3 targeted vaccines in the intervention arm (33.2%) compared with no centralized reminders (29.8%; odds ratio [OR] 1.20, 95% CI 1.06-1.36; *P*≤.01) [38]. Similarly, receiving either culturally tailored or standard electronic messages from primary care providers modestly increased COVID-19 vaccination uptake compared to no outreach from primary care providers (24% and 23.1% vs 21.7%; *P*<.01) [40].

#### Personalized and Tailored Communication Interventions

In total, 7 interventions examined tailored telephone calls to promote preventive services uptake [30,41-43,46,49,52]. Across the 7 interventions, most interventions used low-complexity approaches, primarily relying on manual telephone contact (Table 3). Only 4 of the tailored telephone interventions demonstrated statistically significant improvements in screening rates over usual care.

3 of the studies evaluating tailored or reminder-based interventions found largely nonsignificant improvements in screening uptake overall, though some age-related differences were reported. In one study, investigators used results from a baseline survey on knowledge and barriers to mammography to tailor messages to participants [52]. At the 4-month follow-up, although nonsignificant differences were found among younger participants, older women in the intervention group had significantly higher rates of obtaining a mammogram compared to controls (51% vs 25%) [52]. Luckmann et al [42] compared 2 interventions to a comparison group: a reminder letter with multiple reminder calls or reminder calls with tailored counseling, compared with only a reminder letter. Absolute difference in mammography adherence in the reminder call only intervention group compared to the other arms increased significantly in the 50 to 74 years age range by 3.3% but not in the 75 to 84 years age range [42]. However, the change in mammography adherence from baseline was not significant in either age group for either trial arm [42]. Another intervention to increase colorectal screening compared structured telephone calls after 2 reminder letters to no telephone call [43]. Although overall screening participation was 54.9%, and screening adherence was higher in older age groups than in younger ones (Table 1), there was no between-group difference [43].

**Table 3.** Complexity of technology for personalized and tailored communication interventions.

Study	Targeted preventive care service	Simple intervention description	Technology	Digital intervention reach	Patient-facing complexity
Basch et al (2015) [30]	Colorectal cancer (CRC) screening	CRC print education material, academic detailing to primary care providers, and targeted telephone education for patients	Printed brochures +telephone calls (staff-delivered)	Median 5 calls per patient	Low
López-Torres Hidalgo et al (2016) [41]	CRC screening	Written information, telephone call, or face-to-face meeting delivering CRC screening information; control group received no information	Telephone (voice call)	Not specified	Low
Luckmann et al (2019) [42]	Breast cancer screening (mammography)	Call with tailored education and motivational interviewing (MI)	Telephone (voice call)	Only 23.5% of women were reached and accepted MI	Low
Luque Mellado et al (2019) [43]	CRC screening	Structured telephone interview (scripted calls) to nonresponders after first invitation round	Telephone (voice call)	68.8% (1614/2343) contact rate	Low
Sepucha et al (2023) [46]	CRC screening	Telephone decision-coaching call	Telephone (voice call)	54% (214/399) of intervention arm reached by decision coaches	Low
Venishetty et al (2023) [49]	CRC screening	Mailed FIT^a^ kits with introductory phone calls by a patient navigator or community health worker; there was variability in FIT kits distributed by health clinics	Telephone	Not specified	Low
Wu and Lin (2015) [52]	Breast cancer screening	Tailored messages to personal barriers	Telephone	Not specified	Low

a FIT: Fecal Immunochemical Test.

In contrast to the largely clinically nonsignificant results, 4 of the studies reported significant differences. Basch et al [30] supplemented print education materials with tailored telephone calls aimed at identifying and addressing colorectal screening barriers and encouraging follow-up with a primary care provider. Among those aged 60+ years who had visited a primary care provider during the intervention period, the intervention group was almost 5 times more likely to receive screening compared to print education material alone (36.4% vs 7.7%; *χ*^2^_1_=7.3; *P*=.01) [30]. Similarly, Venishetty et al [49] compared usual mailed FIT kits with an introductory phone call from a patient navigator, who introduced them to the program and its contents. The return rate for the usual care plus introductory call arm was significantly higher (27.8%) than that for the usual care arm (22.8%; *P*=.003) [49]. Another intervention on colorectal cancer screening found that a decision worksheet paired with a call from a decision coach significantly improved screening rates [46]. Despite only half (54%) of the intervention group having been reached by the decision coach, the intervention arm had higher screening rates than the control arm (35% vs 23%; *P*<.001) [46]. Another intervention compared written, telephone, and face-to-face tailored communication with a control group to improve colorectal cancer screening [41]. Interestingly, both written information and telephone communication, but not face-to-face communication, showed significantly greater screening uptake compared to the control group (Table 1) [41].

#### In-Person Interventions Supplemented With Digital Supports

In total, 4 interventions supplemented in-person preventive care education with digital tools [31,33,34,39]. Across these 4 trials, technology use was uniformly simple, with only 2 studies extending into low-moderate complexity (Table 4). In their study, Chan et al [31] found a 3-minute brief health education telephone intervention approximately 1 week before a 3-minute face-to-face health education intervention during participants’ medical appointments increased vaccination uptake by 57% compared to the 9% increase for the control group that received standard of care (promotional leaflets and poster displays). A 3-modal intervention study found that compared to mass-mailing, an in-person conversation was the most effective (OR 24.63, 95% CI 19.28‐31.46), followed by a telephone one-on-one conversation (OR 14.74, 95% CI 10.96‐19.82) [34]. A comparison study of a video-based intervention for influenza vaccination supplemented with a 15-minute small group in-person discussion with medical students, delivered in health care centers to older adults, improved vaccination uptake (10.3% increase in the intervention group and 3.4% in the control group; Table 1) [39]. Conversely, a tablet-delivered, tailored interactive multimedia intervention delivered in person by a lay health worker that provided individualized information based on participant responses did not increase colorectal cancer screening uptake compared with a small-media print intervention or no intervention [33].

**Table 4.** Complexity of technology for in-person interventions supplemented with digital supports.

Study	Targeted preventive care service	Simple intervention description	Technology	Digital intervention reach	Patient facing complexity
Chan et al (2015) [31]	Pneumococcal vaccination	Brief health-education telephone intervention (3 minutes) delivered by nurses before and during the clinic visit	Telephone (voice call)	Not specified	Low
Fernández et al (2015) [33]	CRC^a^ screening	Tailored interactive multimedia intervention delivered on a tablet computer	Tablet-based interactive program	Not specified	Low-moderate
Fleming et al (2018) [34]	CRC screening	One-on-one conversations (in clinic, telephone, or recorded message) to promote FIT^b^ completion	Telephone (voice call)	Not specified	Low
Jiang et al (2022) [39]	Influenza vaccination	Video-led educational intervention	Educational video	Not specified	Low

a CRC: colorectal cancer.

b FIT: Fecal Immunochemical Test.

### Use of Theory

Of the 23 studies, 9 incorporated theoretical frameworks into their intervention development or design. These studies applied a variety of behavioral theories that focused on intentions, beliefs, motivations, readiness, economics, stages of change, and osteopathic principles. The primary purpose of using theory in the design of interventions was to inform the content and framing of messages (Table 5). Among these studies, 6 of 9 reported significant increases in uptake in the intervention groups, with 3 demonstrating clinically important differences. In contrast, only 4 of 13 atheoretical studies reported a statistically significant increase in preventive care uptake.

**Table 5.** Use of theory.

Author (year)	Theory	Theory use	Intervention development description
Fernandez et al (2015) [33]	Fishbein’s integrated model of behavior change	Message content and framing	The study used behavioral theory to identify psychosocial factors influencing colorectal cancer (CRC) screening, which were then targeted in intervention, select behavior change methods, and develop tailored messages or content.
Fleming et al (2018) [34]	Osteopathic principles	Intervention delivery	The study applied osteopathic principles by designing the intervention to integrate patients as partners in their own care through one-on-one conversations, education, and motivational strategies.
Hagoel et al (2016) [36]	Question-behavior effect	Message framing	The 4 base intervention messages (reminders for CRC screening) were manipulated in terms of grammatical form (interrogative vs noninterrogative) and social context reference (yes or no).
Lieu et al (2022) [40]	Behavioral science	Message content	Outreach messages were crafted using behavioral science principles, including social proof, loss aversion, and authority figures.
Luckmann et al (2019) [42]	Precaution adoption process model	Message content and framing	The intervention counselors assessed which of the 5 stages of readiness each woman occupied: unaware, decided against, undecided, planning, or scheduled. Based on this, they delivered scripted and tailored education to specifically address her stage and encourage timely mammograms.
Szilagyi et al (2020) [47]	Health belief model	Message content	The development of portal reminder letter content drew on the health belief model to target key psychological drivers of vaccination, used health literacy principles to create accessible messages, applied behavioral economics to enhance motivation, and integrated patient or expert feedback to optimize content.
Szilagyi et al (2022) [48]	Health belief model	Message content and framing	The portal messages were structured following the health belief model, which focuses on individuals’ perceptions about health risks (like susceptibility to influenza), perceived benefits of action (vaccination), perceived barriers, and cues to action. Messages were also modeled on precommitment and gain-loss. Precommitment messages asked patients if they planned to get the influenza vaccine, aiming to increase the likelihood of follow-through. Gain-loss messages were crafted to emphasize either the positive outcomes of vaccination (gain frame) or the negative outcomes of not vaccinating (loss frame).
Wang et al (2023) [51]	Transtheoretical model—stages of change (SOC)	Message content and framing	The intervention started with a chatbot assessment of each participant’s stage of change regarding their intentions and plans to receive the vaccine. Participants then received a video tailored to their SOC every 2 weeks for 4 sessions. Their SOC was reassessed each time, allowing the intervention to stay relevant as their readiness evolved.
Wu and Lin (2015) [52]	Health belief model	Message content and framing	The telephone counseling protocol systematically began by assessing each woman’s stage of mammography readiness using health belief model constructs (perceived susceptibility, perceived benefits, and perceived barriers), then delivered messages and action steps aligned with her specific needs and beliefs.

## Discussion

### Principal Findings

Overall, the findings from this review demonstrate that digital interventions for promoting preventive screenings and vaccinations in older adults yield mixed results, with effectiveness varying widely depending on the intervention. While certain strategies showed substantial improvements in uptake, the majority of digital or automated reminders (eg, generic text messages and portal alerts) resulted in only marginal or no increases in screening and vaccination rates.Among the interventions reviewed, those incorporating personalized elements, such as tailored telephone counseling or supplementary in-person education, were consistently more effective than impersonal, automated communications. This suggests that while automation can facilitate uptake, human interaction remains critical for promoting preventive health behaviors in older adults.

### Personalization of Digital and Automated Interventions

Digital and automated communication tools, used in nearly half of the studies, can yield statistically significant improvement in uptake of preventive services among older adults. Older adults appear to respond well to generic approaches that notify and send reminders regarding their preventive activities and screenings. However, the effectiveness of simple, generic reminders is generally limited. These outcomes align with earlier systematic reviews and behavior change literature, which also report modest behavior change impacts from brief digital interventions, especially when applied generically to the older adult population [53].

Evidence from this work suggests that interventions incorporating personalized elements, such as tailored telephone counseling or supplementary in-person education, are generally more effective than impersonal, automated communications. This echoes findings from studies on preventive services and digital health adoption, which suggest that human interaction, particularly when delivered by a trusted source, such as a primary care provider, is a critical facilitator of successful behavioral change in older adults [54,55]. The preference for messages from known, trusted sources and for personalized content reflects broader trends in health communication and aligns with user preference studies, which show higher acceptability for content that has a connection to both their care and provider [56,57]. Further, studies have identified the personalization of messages to be an important condition for patient engagement [56,57].

Despite the importance of tailoring interventions to the user’s context and to engage them effectively [58], there are challenges related to personnel cost, time, and effort with this approach. However, emerging evidence suggests that artificial intelligence (AI) may offer scalable solutions by automating personalization and tailoring at a scale for low cost [59]. One opportunity, such as AI chatbots, is able to automate interactions while also simulating interactive and empathic dialogue [60,61]. Chatbots trained with motivational interviewing techniques have shown promise in supporting smoking cessation [60,62], addressing vaccine hesitancy [61], and promoting behavior change [63]. However, AI-driven personalization carries important limitations and risks: models can amplify existing biases and worsen inequities if trained on unrepresentative data, produce clinically inaccurate or outdated recommendations without continuous validation, and generate “black box” outputs that are difficult to interpret or explain [64]. Additional research is needed to evaluate the comparative effectiveness, acceptability, and ethical implications of AI-based personalization in diverse populations.

### Technology Complexity and Appropriateness

The appropriateness of digital health interventions for older adults is strongly influenced by technology complexity, digital literacy, and the availability of support [65]. While most studies included had low complexity, a consistent limitation across moderately complex digital interventions was the lack of attention to training needs, technical support, or onboarding required for older adults to effectively use digital health platforms such as patient portals, SMS text message reminders, or email notifications. The assumption of a baseline digital fluency may inadvertently perpetuate ageism and disadvantage those with weaker digital skills, compounding barriers to access and potentially widening health disparities [15]. Studies that evaluated engagement metrics frequently reported low rates of message opening or system use, with only about half of older adult recipients successfully engaging with portal messages or receiving SMS text message reminders, highlighting a significant constraint on the intervention’s reach. One recent survey has shown that while many older adults prefer patient portals and email, substantial proportions still rely on traditional methods (hospital websites and telephone calls), with significant numbers excluded by digital-only approaches, especially those aged 75+ years [55]. However, there is an increasing demand for patient portal communication in the population aged 65+ years [66]. This suggests that multimodal, accessible communication is critical for maximizing engagement in older populations.

### Use of Theory

Notable in the digital health intervention studies included in this review was the use of theory in intervention design. Approximately half of the included studies explicitly referenced theory, most commonly behavior change, primarily to inform the content and framing of message delivery. While theory use was described in 9 studies, the depth of integration in intervention design is not explicit in all study descriptions. This is similar to critiques raised in a related cardiovascular preventive care research study, where the rigor and depth of theory application were often unclear or inadequately reported, making it difficult to determine whether theory guided the overall intervention architecture or was limited to message wording [67]. This risk of “surface-level” use of theory, where theoretical constructs inform only minor aspects, such as phrasing or framing, rather than the full intervention pathways, has been documented as an ongoing issue in behavioral health research [68,69]. Fewer than half of published behavior change interventions explicitly cite a guiding theory, making it difficult to determine how theory actually contributed to intervention outcomes. The use of theory to guide the studies would have afforded greater explanatory value to the findings, in particular, offering insights into the largely mixed findings that were observed.

### Limitations

This review has several limitations that warrant consideration. The diversity of study designs, populations, and intervention types prevented meta-analysis, and findings were instead synthesized narratively. While this approach allowed for exploration of patterns across varied contexts, it limits the ability to quantify pooled effects. Data extraction was conducted by a single reviewer and verified by 2 others, which may introduce bias, as dual independent extraction is considered best practice [21]. Many studies lacked consistent control or comparison groups, and several used complex, multicomponent interventions, making it difficult to isolate the contribution of individual digital modalities. Although all included studies had a mean age of at least 60 years, many were not specifically designed for older adults and also targeted younger age groups. Finally, the review was limited to peer-reviewed, English-language publications, which may have excluded relevant findings from non-English–speaking regions.

### Conclusions

While digital communication tools can support modest increases in preventive care uptake among older adults, their effectiveness appears to depend on the degree of personalization. This systematic review’s findings highlight that automation alone may be insufficient and that interventions facilitating patient engagement or combining digital outreach with practical supports may be more likely to achieve meaningful improvements in screening and vaccination rates.

## Supplementary material

10.2196/83446 Multimedia Appendix 1 Embase search strategy.

10.2196/83446 Multimedia Appendix 2 Technology complexity assessment criteria.

10.2196/83446 Checklist 1 PRISMA checklist.

## Abbreviations

AI: artificial intelligence
FIT: Fecal Immunochemical Test
OR: odds ratio
RoB 2: Risk of Bias 2
ROBINS-I: Risk of Bias in Non-randomized Studies—of Interventions
