# Supplementary material for: The Effectiveness of Digital Interventions to Increase Preventive Care Uptake in Older Adults: Systematic Review
Source: JMIR Aging. 2026 Apr 29;9:e83446. doi: 10.2196/83446 (PMC13127855; doi:10.2196/83446)
Supplement: Multimedia Appendix 1 [file aging-v9-e83446-s001.docx]

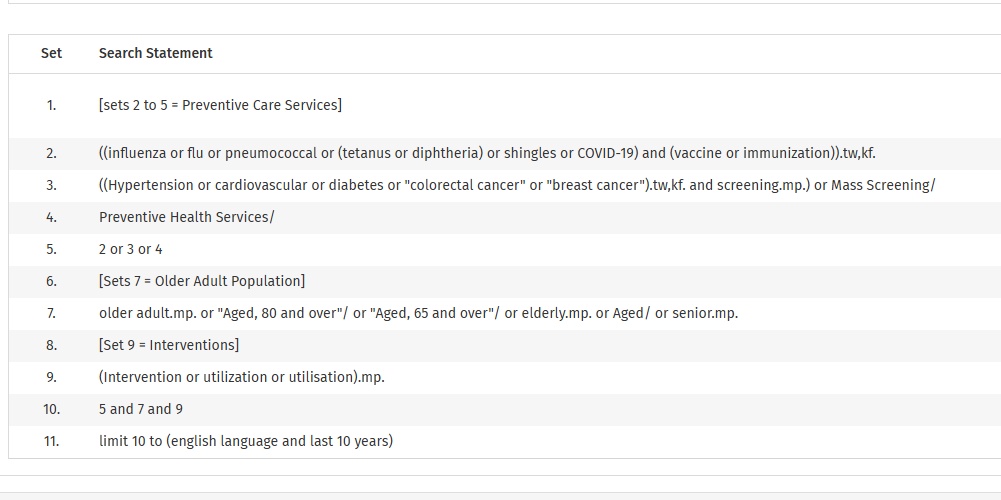


Note: The search strategy was identical across all databases (Embase, MEDLINE, CINAHL, PsycINFO), using the same terms and Boolean operators.
