# Supplementary material for: The Effectiveness of Digital Interventions to Increase Preventive Care Uptake in Older Adults: Systematic Review
Source: JMIR Aging. 2026 Apr 29;9:e83446. doi: 10.2196/83446 (PMC13127855; doi:10.2196/83446)
Supplement: Multimedia Appendix 2 [file aging-v9-e83446-s002.docx]

| **Rating** | **Assessment criteria** |
| --- | --- |
| Low | Minimal interaction required. Users engage passively, for example, reading text messages or emails, or answering a phone call |
| Low-moderate | Requires basic interaction with technology, such as straightforward navigation of simple screens or menus |
| Moderate | Involves multi‑step interactions, such as logging into a portal, reading information, and making decisions. May require two‑factor authentication. |
| High-moderate | Requires completing more complex tasks, such as creating accounts within app‑only environments. Involves interacting with interfaces that frequently change or require active input (e.g., filling forms, submitting information) |
| High | Requires complex digital tasks such as exporting or importing files, connecting multiple devices, navigating multiple applications, and completing advanced authentication steps. For example, downloading data from a wearable device and uploading it to a portal. |
